# BRNI: Modular analysis of transcriptional regulatory programs

**DOI:** 10.1186/1471-2105-10-155

**Published:** 2009-05-20

**Authors:** Iftach Nachman, Aviv Regev

**Affiliations:** 1 FAS Center for System Biology, Harvard University, Cambridge, MA 02138, USA; 2 Broad Institute of MIT and Harvard, Cambridge, MA 02142, USA; 3 Department of Biology, Massachusetts Institute of Technology, Cambridge, MA 02142, USA

## Abstract

**Background:**

Transcriptional responses often consist of regulatory modules – sets of genes with a shared expression pattern that are controlled by the same regulatory mechanisms. Previous methods allow dissecting regulatory modules from genomics data, such as expression profiles, protein-DNA binding, and promoter sequences. In cases where physical protein-DNA data are lacking, such methods are essential for the analysis of the underlying regulatory program.

**Results:**

Here, we present a novel approach for the analysis of modular regulatory programs. Our method – Biochemical Regulatory Network Inference (BRNI) – is based on an algorithm that learns from expression data a biochemically-motivated regulatory program. It describes the expression profiles of gene modules consisting of hundreds of genes using a small number of regulators and affinity parameters. We developed an ensemble learning algorithm that ensures the robustness of the learned model. We then use the topology of the learned regulatory program to guide the discovery of a library of *cis*-regulatory motifs, and determined the motif compositions associated with each module.

We test our method on the cell cycle regulatory program of the fission yeast. We discovered 16 coherent modules, covering diverse processes from cell division to metabolism and associated them with 18 learned regulatory elements, including both known cell-cycle regulatory elements (MCB, Ace2, PCB, ACCCT box) and novel ones, some of which are associated with G2 modules. We integrate the regulatory relations from the expression- and motif-based models into a single network, highlighting specific topologies that result in distinct dynamics of gene expression in the fission yeast cell cycle.

**Conclusion:**

Our approach provides a biologically-driven, principled way for deconstructing a set of genes into meaningful transcriptional modules and identifying their associated *cis*-regulatory programs. Our analysis sheds light on the architecture and function of the regulatory network controlling the fission yeast cell cycle, and a similar approach can be applied to the regulatory underpinnings of other modular transcriptional responses.

## Background

**Figure 1:**
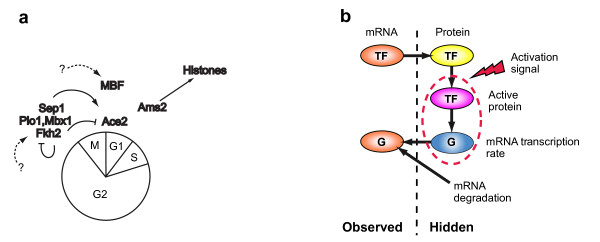
**Modeling Transcriptional Regulation**. (**a**) The *S. pombe *cell cycle transcriptional regulatory program. Shown are the phases of the cell cycle, known regulators and their regulatory interactions (arrows – activation; blunt arrows – repression). Figure adapted from [9], with some additions. (**b**) A qualitative molecular model of transcriptional regulation. mRNA encoding a transcription factor (TF, orange oval) is translated to protein (yellow oval). The protein is activated (pink oval) and induces the transcription of a target gene at a certain rate (G, blue oval). The final accumulation of G mRNA levels (G, orange oval) is determined by this transcription rate and by the rate of G's mRNA degradation. Each of the ovals is associated with a relevant quantity (TF mRNA level, TF protein level, activated TF protein level, transcription rate of the target gene G and mRNA level of G). A microarray experiment only measures the first and last of these quantities ("observed"), whereas the other quantities are not observed ("hidden"). The dashed oval encloses the closest quantities on this path between the TF and the target gene G. Our approach models the connection between these two variables.

Despite the major role of regulatory networks in orchestrating complex cellular functions, the architecture and function of most networks is largely unknown. Several methods were previously suggested for reconstructing the structure of regulatory networks from expression data. Most methods learn simplified models [1-5] based on abstract regulator-target relations rather than a biochemical model of the binding of a transcription factor (TF) to a promoter. Furthermore, since they rely on the mRNA levels of both target *and *TF, they fail when the TF is not itself regulated at the transcription level (Figure 1b).

Complementary approaches learn a regulation program by integrating gene expression data with additional data sources, such as genome-wide TF binding data [6] or promoter sequence information [5,7,8], into a single coherent model. Each of these approaches has some limitations. TF-binding data are still scarce, can suffer from high false positive rates, and even true binding of a TF does not necessarily imply regulation. Analyzing promoter sequences is limited by the relatively small number of known *cis*-regulatory motifs, the difficulty to detect significant novel binding motifs, and the high false positive rate when scanning for motif occurrences in promoters. Nevertheless, by requiring consistency between several heterogeneous data types, integrative models are typically more robust and accurate.

Here, we present a novel integrated approach to analyze transcriptional regulatory programs. We use a gene expression data set to decompose genes into coherent modules of co-regulated genes, based on a biochemically-motivated model. Our model uses realistic constraints, suggesting a mechanistic explanation for their expression patterns using combinations of a small number of unknown putative regulators. We employ two novel strategies to increase model robustness. First, we use gene modules – sets of targets controlled by the same biochemical regulatory functions – to learn a global network model which is simpler and biologically meaningful. Furthermore, we devise an algorithm that learns a robust model based on an ensemble learning approach. Although the biochemical constraints are insufficient to build a fully realistic model with current datasets, they provide a principled way to extract a biologically coherent modular structure for the data.

We then use this modular decomposition to search for novel binding motifs in sets of genes defined by the network structure, and test for enrichment for those motifs in all the learned modules. The motif combinations present in the target genes define a second, sequence-based regulation program. In particular, it allows us to explore the regulation of transcription factors.

We apply our approach to the transcriptional program of the fission yeast cell cycle, a system which is only partially characterized [9]. In particular, a large portion of the cell cycle (G2), and the transition from G2 to M are not explained by any known transcriptional regulator (Figure 1a) in fission yeast. This is in contrast to the regulatory program of the cell cycle of the budding yeast, *Saccharomyces cerevisiae*, where a closed loop of transcriptional regulators is known [10]. Previous studies show that one cannot project the regulatory program from budding yeast to fission yeast: the set of regulators is only partially overlapping between these two divergent species, as are their target gene set and binding site sequences (reviewed in [9], see also [11]). This is consistent with the functional differences between the cell cycle of these two species (*e.g*. strikingly different duration of the different phases). Several studies measuring genome-wide expression profiles in *S. pombe *throughout the cell cycle were recently published [12-14], but their initial analysis only partly filled up the gaps in understanding.

Our analysis discovered 16 coherent modules spanning different phases of the cell cycle and covering diverse processes from metabolism to cell division. The resulting learned motif library is composed of 18 regulatory elements, including both known cell-cycle regulatory elements and novel ones. Finally, we analyze how specific regulatory topologies underlie distinct dynamics behaviour of gene expression in the fission yeast cell cycle.

## Results and Discussion

**Figure 2:**
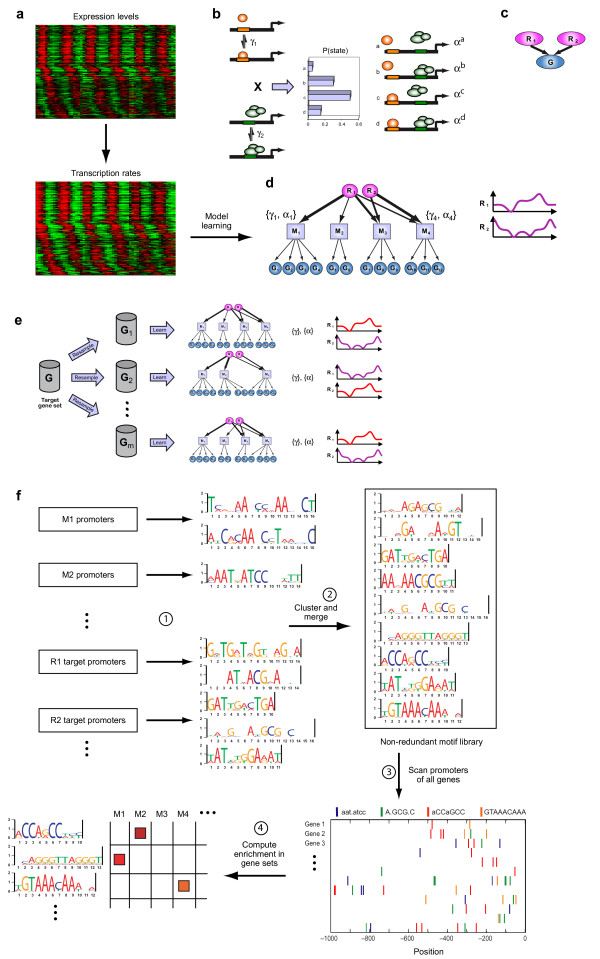
**Flow of the integrated analysis**. (**a-d**) **Learning a biochemically based regulatiozn model. **The input for model learning is transcription rates derived from mRNA levels (**a**). A biochemical model of TF binding and dissociation (**b**) is used to describe the transcription rate of a target gene. The binding and dissociation kinetics of each transcription factor (orange and green ovals) to the target gene promoter (left panel) are governed by affinity parameters (γ_1 _and γ_2_, respectively). These kinetics result in a distribution of promoter states within the cell population (middle panel). Each promoter state is associated with a distinct transcription rate (α^a ^through α^d^, right panel). These regulation functions are used within a probabilistic graphical model (**c**) where the observed transcription rates of a target gene (G, blue oval) are explained using the hidden active protein levels of the regulators (R1 and R2, pink ovals). In practice we learn a modular model (**d**), where the genes belonging to a single module (square nodes) share the same set of affinity and transcription rate parameters {**γ**, **α**}. The model topology describes which regulators control each of the modules, and which genes are members of each module. In addition, the regulator activity profiles (right) and all kinetic parameters are inferred. (**e**) **An ensemble learning approach. **From the original set of genes (G, barrel), *m *subsets (G_1 _through G_m_) are randomly sampled, each containing some fraction (*e.g*. 80%) of the genes. A modular regulation model is learned for each subset as in (**d**). The resulting ensemble of models is integrated into a unified consensus model (Methods). First, regulators are mapped between different runs based on their time profile similarities (e.g. red profiles on right panel). Next, core gene modules are defined based on sets of genes that frequently co-occur in the same module. (**f**) **Learning a motif-based regulation model. **Subsets of genes are defined either by members of a module, or by targets of a regulator in the unified model. The promoters of these gene subsets are searched for novel *cis*-regulatory motifs using four different algorithms. The resulting redundant collection of motifs is clustered and merged to generate a non-redundant library of motifs. The promoters of all genes are then scanned against this library, and enrichments of gene sets for particular motifs are computed.

We developed an integrated approach to analyze the regulatory program controlling gene expression during a dynamical process from expression and sequence data (Figure 2). We illustrate and test the steps of our approach based on the fission yeast cell cycle dataset. Our approach consists of six steps: (**1**) We derived an **input set **of transcription rate profiles for 248 cell-cycle regulated genes from a gene expression time series data set spanning 6 cell cycles [12] and sampled 90 datasets from this input set, each containing 200 genes. (**2**) We learned a **regulation model **for each of the 90 data sets. Each such model identifies a set of **modules**, co-regulated target gene sets, and describes their transcription rate profiles using a set of learned regulator activity profiles and a set of regulator-to-gene affinity parameters. (**3**) We generated a **unified model **from the resulting ensemble of 90 models, capturing the variance and significance of different elements in the individual models. (**4**) We used the structure of the unified model to guide a search for novel *cis*-regulatory motifs, resulting in a **library of 18 motifs. **(**5**) We identified the **motif composition **of each promoter by scanning the promoters of all *S. pombe *genes against our library and identified motifs enriched in core gene modules in our unified model. (**6**) We contrasted the expression- and sequence-based regulatory relations, highlighting key elements of transcriptional regulation in the *S. pombe *cell cycle. Below we describe each step of our approach.

### Biochemical Regulatory Networks: an expression-based biochemical model of modular gene regulation

We developed a novel algorithm, Biochemical Regulatory Network Inference (BRNI), which takes expression levels for a set of genes, converts them to transcription rates (Methods) and learns a biochemical model of gene regulation. In our modular regulatory model, inferred regulators are connected to modules of co-regulated genes (Methods, Figure 2d), and control their dynamic behaviour based on biochemical principles (Figure 2b).

BRNI is based on our method to infer biochemical models of single gene regulation [15]. This method *infers *a set of regulators needed to explain the observed expression levels, and for each such regulator it learns a temporal activity profile *r*_*j*_*(t)*, representing its activity levels over time. The connections between (inferred) regulators and (observed) target genes follow biochemical rules that describe how the regulator controls the expression of the gene, based on affinity parameters (Figure 2b). The model accounts for the biochemical processes of binding and dissociation, thus allowing for different non-linear combinations of regulators, both as activators and as repressors. Specifically, for each gene the learned biochemical model includes the set of regulators controlling it (one or two), the affinity parameter *γ*^*j*^_*i *_between the gene and each of its regulators, and the gene-specific activity levels of each binding state {***α***_*i*_}. The set of affinity and activity parameters {*γ*^*j*^_*i*_, ***α***_*i*_} uniquely defines the target gene transcription rates as a function of the regulator behaviour *r*_*j*_*(t)*. A multiplicative noise model is used to account for deviations between observed transcription rates and those predicted by the regulation functions.

Such a detailed biochemical model contains, however, many parameters (up to six for each target gene). Given the limited amount of data, the learned model might represent over-fitting of this data. In particular, it can be strongly biased by data points or genes suffering from high measurement errors. To overcome this, we developed here two novel and complementary approaches: **(1) **modifying the model to include modules of target genes; and **(2) **using a bootstrap approach, where we learn an ensemble of models from which we derive a high-confidence unified model.

First, we modified our model to introduce ***target gene modules***. Each module consists of genes with similar expression patterns, the same set of inferred regulators and the same affinity and activity parameters (Figure 2d). This greatly reduces the number of parameters in the learned models and lowers the dimensionality of the search space, thus increasing the model's robustness and speeding up the search. We devised an iterative search algorithm that learns this modular model by alternating between refinement of the module regulation model (association of regulators to modules, splitting/merging of modules, optimization of regulation parameters) and optimal assignment of genes to modules (Methods).

Second, we devised a ***bootstrap procedure ***for learning a model: rather than learning a single model, we learn an *ensemble *of models each based on a different sampled subset of target genes (Figure 2e). The speedup gained by the model's modularity allows us to learn an entire ensemble of models in reasonable time.

Finally, we integrate the ensemble of models into a ***unified consensus model ***(Methods, Figure 2e). We first *map the regulators *between different runs based on their time profile similarities. Next, we define *core gene modules *based on sets of genes that frequently co-occur in the same module. In the resulting integrated network each regulatory connection is assigned a confidence score, and each affinity parameter is associated with an error bar.

### Learning regulatory modules in the fission yeast cell cycle

**Figure 3:**
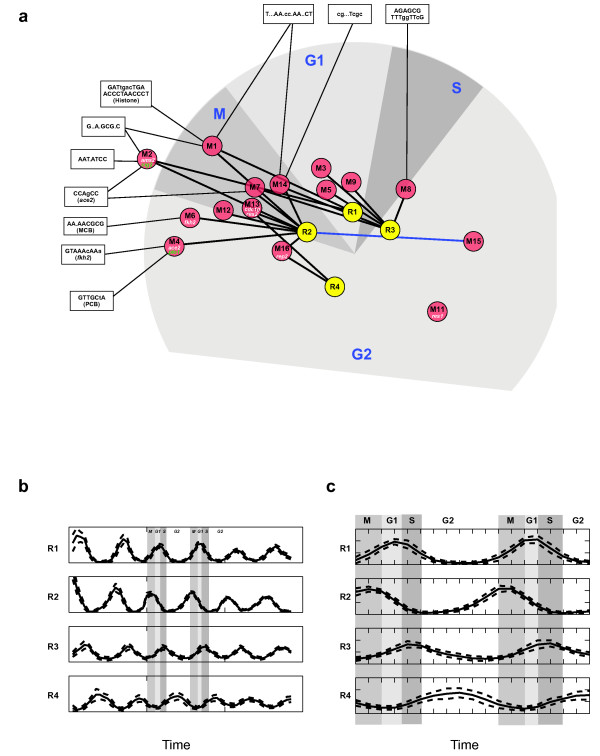
**An integrated model of transcriptional modules in *S. pombe *cell cycle**. (**a**) A map of the unified model topology. Shown are fifteen modules (red nodes) and four regulators (yellow nodes) and their regulatory connections (thick edges) along the *S. pombe *cell cycle. The angular position and the radial distance of each module node represent the respective average peak phase and the average amplitude of transcription rates among the module members. The angular position of each regulator node represents the peak phase of its activity profile. Known cell cycle regulators that could be associated with a particular module (as members) are denoted within the module node (transcription factors – white; kinases – green). The blue edge signifies a repressive regulatory connection, while all the other connections are activatory. The thin edges connect modules with binding motifs that are significantly enriched in the module's promoters (see Additional file 3). (**b**) Inferred activity profiles of the four regulators R1-R4 in the unified model. Mean and one standard deviation curves are shown. (**c**) Zoom-in of the middle time series (Elutriation 2) in (**b**).

We applied our algorithm to expression profiles of 248 cycling genes measured during the fission yeast cell cycle [12] and derived a unified model with 4 regulators controlling 16 core modules (Methods, Figure 3, Table 1, Additional file 1). Our analysis shows that both the initial structure learning, as well as the bootstrap step improve the coherence of the resulting modules (see Additional files 2, 4, 5, 6, 7, 8, 9 and 10). The modules consisted of 7 to 27 genes with correlated expression profiles and a distinct phase (the only exception is Module #10, that contains 11 noisy genes). Five modules (containing 77 genes) peak at M/G1; two modules (42 genes) peak at G1; two modules (25 genes) at G1/S, two modules (22 genes) at S/G2 and four modules (78 genes) at G2. Several of the distinct modules represent coherent biological processes, as reflected by their members' known functions (Methods, Table 1). The modules cover both classical cell cycle processes (*e.g*. Histone genes in Module 1, cell wall and cell division genes in Modules 2 and 4, spindle formation and cell polarity in Module 12) as well as general growth processes (*e.g*. metabolism genes in Module 3, translation regulation and ribosome biogenesis in Module 8). Each of the four regulators, denoted R1 through R4, has a distinct cell cycle phase (Figure 3b, c). R2 peaks at G2/M and is the dominant regulator in the model. R1 peaks at G1, R3 peaks at G1/S and R4 has a wide peak at G2.

**Table 1:** List of 16 core modules defined by the ensemble.

**Module Number**	**Main functions**	**Expression peak phase**	*Transcription peak phase	**Number of genes**	**Member genes that are known cell cycle regulators (TFs in bold)**
1	Histones (7/7)	S	M/G1	7	

2	Cell wall (6/12); glycoproteins (3)	M/G1	M	12	cig2,**ams2**

3	Metabolism (6/12)	S/G2	G1/S	12	

4	Cell wall (4/11), cell division (4/11)	M/G1	G2/M	11	**ace2**, plo1, cdc15, slp1

5	Mixed	S/G2	G1/S	10	

6	Cytokinesis (2/9);	M/G1	G2/M	9	**fkh2**

7	Mixed	G1	M	27	

8	Translation regulation (9/20) (ribosome biogenesis (7/20)); transporters (4/20)	G2	G1/S	20	

9	Mixed	G2	G1/S	15	

10	Mixed	Mixed	Mixed	11	

11	Mixed	G2/M	G2	15	

12	Mixed	G1	M	15	pmk1

13	Mixed	M/G1	M	18	**cdc10**, **res2**, csk1

14	Mixed	G1/S	G1	18	

15	Cell wall (6/21)	Early G2	S/G2	21	

16	Mixed	M	G2	27	cdc13, crk1, cdr1, clb1

The 16 gene modules in the unified model. For each enriched function, the number of genes in the module having that function is shown in parentheses. *Transcription rates, as predicted by our pre-processing (see Methods).

### Learning a motif-based model of gene regulation

A complementary view of the transcriptional program is driven by the promoter sequences of the target genes. Assuming a transcription factor binds a specific motif, the full motif set in the promoters of the target genes induces a connectivity model between TFs and target genes. The structure of our expression based learned network therefore provides two key clues to finding *cis*-regulatory motifs. First, we can search for motifs enriched in a module, as the co-expression of module genes may indicate a shared regulatory mechanism. Second, we can also search for motifs shared by the targets of the same regulator across modules. If that regulator corresponds to a DNA binding factor, or even to an indirect regulatory activity, we expect its targets to share a regulatory element (Figure 2f). Importantly, these targets can be distributed across multiple modules with distinct expression patterns due to combinatorial regulation. Thus, such related motifs may not be identified by the former, module-based approach. Notably, a comparative analysis of different partial models shows that different components of the regulatory model (modular structure learning and ensemble learning) improve the resulting modules in terms of their correspondence with known binding motifs (Additional files 2, 4, 5, 6, 7, 8, 9 and 10). This suggests that the same model can also improve the discovery of novel motifs.

We used an automated approach (Methods), to systematically learn a non-redundant **library of motifs **in this manner. The resulting library consists of 18 motifs, 14 of which were derived from modules and 4 from regulators. These motifs match the known cell cycle regulatory elements MCB (bound by the MBF complex), Ace2, Fkh2 (FLEX motif), PCB and the histone ACCCT box, as well as include several novel motifs (Additional file 3).

We next scanned the whole genome against this library and tested each motif for enrichment in the promoters of gene module members. We found 18 significant motif-module pairs. Seven of the modules are significantly enriched for at least one motif (Additional file 3, Figure 3). Notably, we found no enrichment when we performed a similar scan of *S. pombe *modules with five additional known cell-cycle motifs from *S. cerevisiae *that do not have a known counterpart in *S. pombe *(MCM1, YHP1, YOX1, ASH1 and FHL1 [16]). This is in contrast to the above mentioned elements MCB, Fkh2 and Ace2, which are similar or identical to their *S. cerevisiae *counterparts.

### cis-regulation of expression modules in the fission yeast cell cycle

The motif analysis resulted in several interesting insights on the regulatory mechanisms controlling each module some of them recapitulate known facts, indicating the validity of our results, while others are novel, and suggest new testable hypotheses. For example, the **Histone Module (#1) **consists of all seven histone genes in the input set (two other *S. pombe *histone genes were excluded from the input set due to multiple missing values). The transcription rates of the module's genes are predicted to peak at M/G1, while their measured expression levels peak at S phase. The genes in this module are associated with only four promoters, since eight of the nine *S. pombe *histone genes are arranged in divergently-transcribed pairs. We found that all histone promoters contain a previously described histone specific motif (AGGGTTAGGGT). Recent studies show that this site is bound and activated by the Ams2 transcription factor [17]. Our analysis also shows that Ams2 itself is a member of Module 2, and has an MCB motif in its promoter, and another novel motif, A.GCG.C. Interestingly, two of the histone promoters contain an MCB site as well, and three of them contain the A.GCG.C motif. This suggests a possible feed forward loop involving the MBF complex (that binds MCB), Ams2 (in module 2) and the histone genes (in Module 1), as we discuss below. The regulation of histones by MBF may be a conserved feature of the yeast cell cycle transcriptional network. In *S. cerevisiae *the promoters of histone genes contain mostly Swi6, Swi4 and Mbp1 motifs, raising the possibility of their activation by MBF and/or SBF [10]. Finally, we discovered a third novel motif (GATtgacTGA) that appears in three of the four promoters. This motif might serve as the (unknown) binding site for the repressor Hip1. Further experiments are needed to validate the proposed regulatory role of MBF and the novel sites in *S. pombe *histone genes.

**Figure 4:**
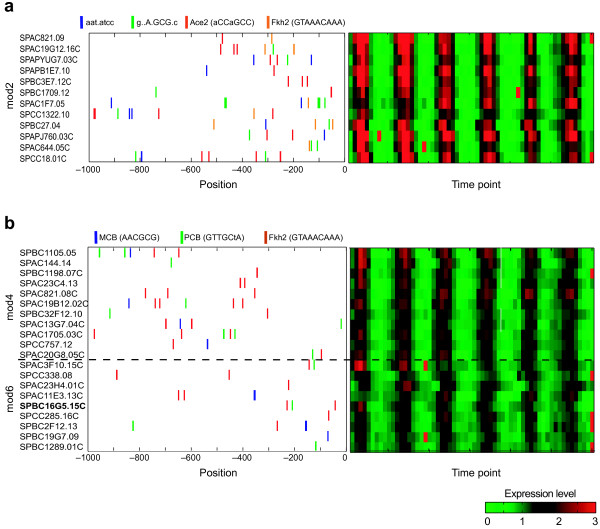
**Promoter composition vs. expression profiles of module genes**. Shown is the promoter composition of genes in a module (left panel) along with the expression profiles of the corresponding genes (right panel). Each row represents one gene, where gene names are shown on the left. Binding sites for selected motifs are denoted by color bars, while position is denoted as distance from ATG. (**a**) Module 2 (**b**) Module 4 and Module 6.

The **Cell Division Module (#2) **consists of 12 genes, encoding mostly cell wall proteins and glycoproteins whose expression peaks at M/G1. The module genes' promoters are enriched for the Ace2 motif (9 genes), the Fkh1/2 motif (6 genes), and for two novel motifs (AAT.ATCC in 7 genes and A.GCG.C in 8 genes, Figure 4a). Regulation of the module by Ace2 is consistent with the module's function (cell division), the down-regulation of nine module genes in an *ace2 *deletion strain [12], and the phase of Ace2 transcription which slightly precedes that of the module's genes, consistent with a positive regulatory role. Further experiments are needed to explore the additional role of Fkh2 and the factors binding the novel motifs in modulating the expression pattern of the module's genes.

The two **cell wall biogenesis and cell division **modules (**#4 and #6**, 20 genes) include the Ace2 and Fkh2 genes. The modules' genes are associated with a putative PCB motif (GTTGCTA, 11/20 genes) and a Fkh2 motif (18/20 genes, multiple sites per promoter). Although the phase of these modules is similar to that of Module 2, their genes do not contain any Ace2 sites, supporting their separation to distinct modules. Notably, the Fkh2 sites in module 4 are concentrated further upstream of the gene start than in module 6 (Figure 4b), supporting their further separation. The hypothesis that Fkh2 and Sep1 (which binds PCB sites) are joint regulators in these modules is supported by the effect of sep1 deletion on several of the modules' genes [12] and by the similarity of the peak phase of rate of transcription of the modules' genes and Fkh2 (both at G2/M). These results are consistent with a recent study [18] demonstrating that in three promoters containing both sites, both Fkh2 and Sep1 bind and play opposing roles in repression and activation of their joint targets, respectively. Notably, 7 of the 20 modules' genes have only Fkh2 sites in their promoters, suggesting that Fkh2 can regulate expression in promoters that lack PCB. Since only a repressive role has been demonstrated for Fkh2 in *S. pombe*, these promoters may be regulated solely by de-repression or by a novel unknown mechanism.

The **Translation Module (#8) **consists of 20 genes, mostly related to translation regulation and ribosome biogenesis that peak in early G2 phase, the major growth phase for *S. pombe*. The module contains two prominent yet unknown motifs – AGAGCG (11 genes) and TTTggTTcG (8 genes). Each of these motifs appears in approximately 5% of all *S. pombe *genes, and is enriched in genes that perform metabolic functions. Since the expression of genes encoding the translation and ribosome biogenesis machineries is also modulated in response to environmental stresses, the discovered motifs could be responsible for their regulation either under stress or in normal cell cycle conditions. In the latter case, they could provide a novel mechanistic explanation for cell cycle regulation during the G2 phase.

Overall, the analysis led to several testable hypotheses on the fission yeast cell cycle: **(1) **MBF and Ams2 form a feed-forward loop to control histone gene expression; **(2) **Histone gene expression is controlled through the novel motif GATtgacTGA, which may be a Hir1 target site; **(3) **Fkh2 may control the cell division module; **(4) **Fkh2 may control distinct modules involved in cell wall biogenesis and cell division, both in combination with Sep1, and alone, possibly solely though a de-repression mechanism; and **(5) **Growth related functions, such as ribosome biogenesis, are under cell cycle control through two novel *cis*-elements, AGAGCG and TTTggTTcG.

### The power and limitation of an expression based regulatory model

We next compared the expression- and *cis*-regulatory networks we learned. In particular, we examined whether learned regulator profiles correspond to specific transcription factors. If this is the case, we expect each regulator to be mapped to a specific regulatory element from our library.

When considering each of the regulators, however, we do not find such matching. For example, the regulator **R2**, peaks at G2/M and captures a "centralized" activity around the narrow time interval covering M/G1, G1 and G1/S, and is thus associated with the regulatory elements and activity of several transcription factors active during those phases (Ace2, MCB, Fkh2 and others, see analysis above). Similarly, **R1 **peaks at M/G1, and is connected to the many histone sites through Module 1 genes, but also to Ace2 motif (through Module 2 genes). Its combination with R2 explains the delayed activity of this module's members. Overall, we find that the learning algorithm avoids the need for additional regulators to explain the expression of different modules in those phases by using either **R2 **alone or in different combinations with the other learned regulators, thus achieving more delayed or early expression peaks.

Although the individual learned regulators do not correspond to specific transcription factors, the network induced by their combinations is meaningful. First, as discussed above, the network consists of modules with coherent biological functions. Second, the network topology allowed us to discover most known cell cycle binding sites and several novel ones. Third, the modules display distinct binding site compositions. In particular, in several cases (*e.g*. Modules 2, 4, and 6 discussed above) genes with very similar expression profiles were partitioned into separate modules. Our analysis showed that each of these modules was characterized by a distinct promoter configuration, supporting the partition. This strength of the regulatory model is due to its non-linear nature. Future work can incorporate motif finding and scoring as an integral part of the learning algorithm, thus using cis-regulatory distinctions to identify concrete regulators.

### Reconstructing a network of transcriptional regulation

To further associate the inferred regulatory networks with concrete regulatory functions, we examined whether the relation between the timing of expression of cell-cycle related transcription factors and the timing of expression of gene modules that are associated with their binding sites. Naively, we would expect to find an activator's binding site in promoters of genes which are induced in a subsequent phase. Conversely, we expect to find the binding sites for repressors in promoters of genes that are repressed in the subsequent phase. This simple prediction may be distorted by a delay between the regulator's gene transcription and the binding of its protein to target promoters. Such a delay could result from slowed dynamics or active regulation in any of the intermediate steps between transcription and binding (*e.g*., translation of the regulator protein, its activation or its localization into the nucleus).

**Figure 5:**
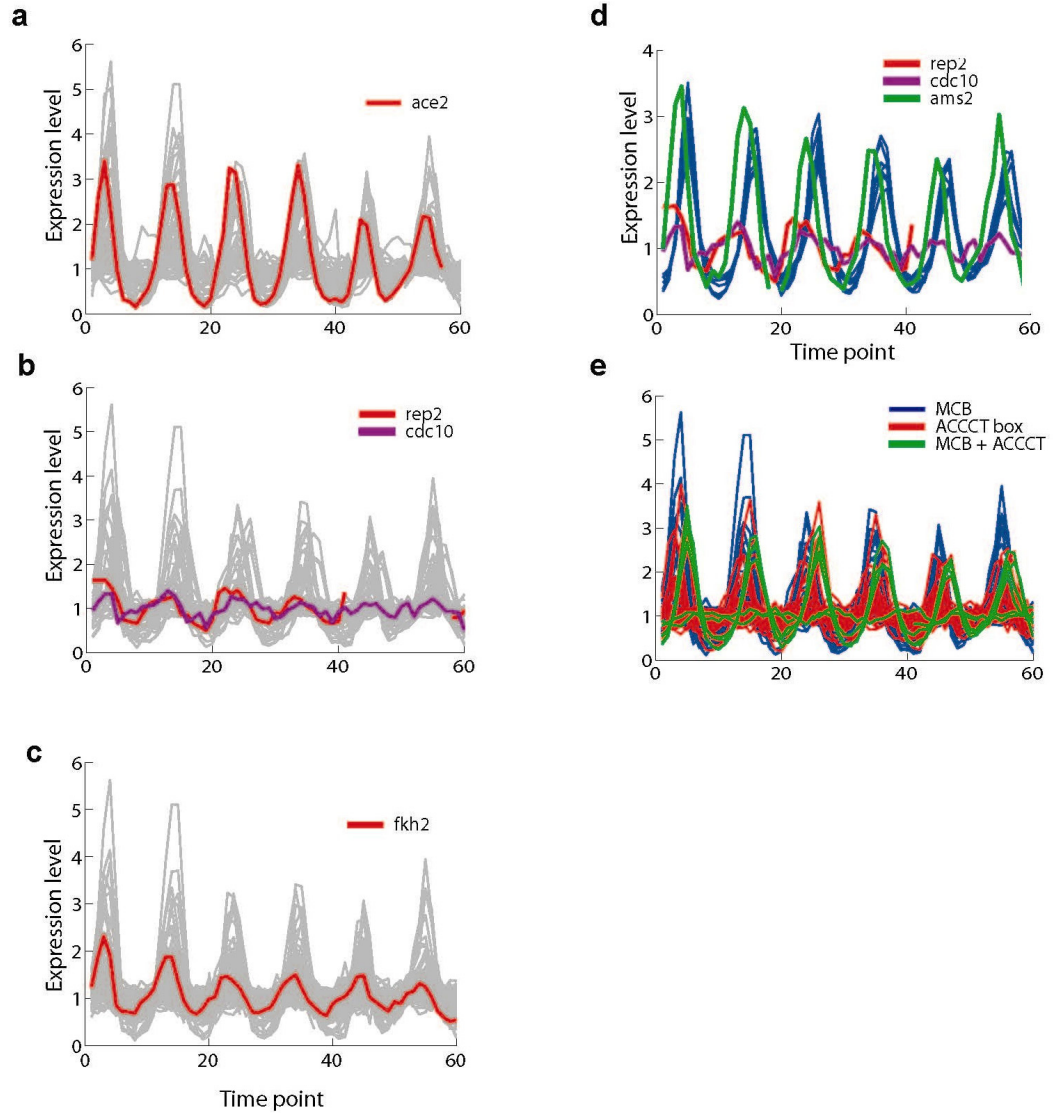
**Coherence of regulator expression with that of its targets**. (**a-c**) Shown are the expression profiles of a transcription factor (red, magenta) vs. the expression profiles of all cycling genes whose promoter contains a binding motif for that factor (light gray). (**a**) ace2; (**b**) MBF (two cycling components are shown); (**c**) fkh2. (**d**) Expression profiles of histone genes in Module 1 (blue), ams2 (green) and MBF components rep2 (red) and cdc10 (magenta). (**e**) Expression profiles of cycling genes containing either an MCB motif (blue), an ACCCT box (red) or both (green).

**Figure 6:**
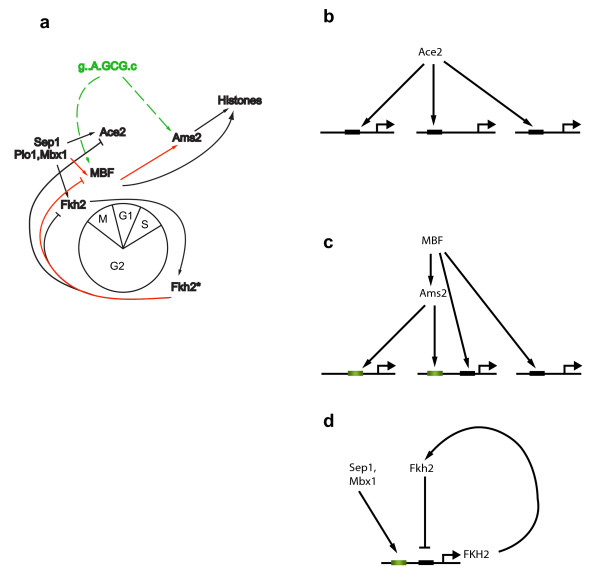
**A transcriptional regulation network for the *S. pombe *cell cycle**. (a) An enhanced model for the transcriptional regulatory network controlling the *S. pombe *cell cycle. New insights or connections are denoted in red. Connections to novel motifs related to unknown regulators are denoted in green dashed lines. Fkh2* denotes Fkh2 bound to its target promoter. (**b-d**) Some of the regulatory motifs found in the cell cycle network. (**b**) Ace2 regulates its targets through a simple direct activation. (**c**) Ams2, controlled by MBF through the MCB motif, binds the ACCCT box [17]. Different genes have different combinations of these two sites in their promoters. Genes that have both MBF and Ams2 sites are part of a feed-forward loop. (**d**) Fkh2 regulates itself through a negative feedback loop, while being activated by Mbx1/Sep1/Plo1 complex.

We examined each of the four cell-cycle transcription factors which have both a cyclic transcriptional profile and a known binding motif: Ace2, Cdc10 and Rep2 (the two cycling subunits of MBF), Fkh2, and Ams2 (Figure 5). We found that each is associated with a distinct mechanism resulting in different dynamic behaviour of its targets, together forming an integrated network with a cyclic behaviour (Figure 6).

First, Ace2's expression slightly precedes that of its targets in Module 2, supporting a simple activatory model (Figure 5a, 6b). Other targets (*e.g*. Module 7) exhibit a longer delay, but their regulation mechanism might be different since they do not respond to an *ace2 *deletion [12].

Second, MBF and Ams2 target genes display a narrow spectrum of peak times, from in-phase with their respective regulator up to a slight delay from that regulator (Figure 5b, d, e). These spectra may be achieved by interactions of these transcription factors with other regulators. For example, genes whose promoters harbour both Ams2 sites (the histone ACCCT box) and MBF sites (MCB) exhibit a delayed (and sharper) expression phase compared to the effect of each one of these regulators alone (Figure 5e). Upon closer inspection, we find that Ams2 is in fact part of a feed-forward activatory chain: it contains an MBF site in its promoter, its expression is slightly preceded by Cdc10 and Rep2, and it precedes the histones' expression profiles (Figure 5d, 6c).

Finally, there is no delay between the peak expression of Fkh2 and the genes in its target modules (Modules 4 and 6, Figure 5c). This may be explained by its repressive role and a delay between its transcription and its binding to target promoters, as recently reported in [18]. This work showed that while Sep1 is likely an activator and binds concurrently with the expression of its target genes, Fkh2 is likely a repressor and binds when the expression of the same target genes is low. Since Fkh2 itself is regulated in this fashion (it is a member of Module 6) it might close a negative feedback loop, suggesting a mechanism for regulating the G2/M part of the cell cycle: Fkh2 is transcribed during M/G1, and following translation and localization to the nucleus binds to these promoters for the length of G2, inhibiting their activation by Sep1. As it degrades gradually during G2, by the end of this phase it no longer prevents Sep1 binding and activation during M/G1 (Sep1 is constitutively expressed).

In conclusion, we propose the following model for the transcriptional regulatory circuit governing the cell cycle, as it emerges from our integrated analysis (Figure 6a). As explained above, Fkh2 is the only component that could by itself close a loop of transcriptional regulation around the cell cycle. Along with the Sep1/Mbx1 PBF complex, it regulates several other regulators, including the MBF subunit Rep2, Ace2 and Ams2. Finally, the motif gA.GCG.c, which is similar to MCB and could be an alternative variant of it, plays a dominant role both in cell cycle regulated genes as well as in the promoters of some of the regulators themselves (Cdc10 and Ams2). Thus, our integrated analysis discovered novel players, interactions and dynamics in the *S. pombe *cell cycle, in particular suggesting how combinatorial regulation can lead to a full cyclic circuit of transcriptional regulation.

## Conclusion

We have presented an integrated approach for the analysis of transcriptional programs. Our analysis comprised of two components: a biochemically motivated model of gene regulation based on the expression data, inducing a division to expression modules; and a binding motif analysis based on the division to those modules and regulatory relations. We have applied our approach to the analysis of the fission yeast cell cycle program. This approach is generally applicable to expression profiles measured along time courses.

The regulation program we learned allowed us to derive important biological insights. First, it induced an informative division to coherent regulatory modules. In particular, it was able to separate between modules with similar expression peak phases but with clearly distinct binding site compositions, based on more subtle differences in expression profiles. This division resulted in identification of several novel binding sites (as well as recapitulating most known cell cycle regulatory motifs) in the second part of the analysis, allowing us to detect the structural features underlying distinct dynamic behaviour.

Our analysis suggests several novel potential mechanisms for differential regulation of genes along the cell cycle. These include a feed-forward chain of MBF with Ams2 leading to delayed expression of histone genes, a putative binding site for the histone gene repressor Hip1, combinatorial regulation of specific cell division genes by Ace2 and Fkh2 (rather than by each factor alone), putative sites acting during the G2 phase to regulate cell growth modules, and a negative feedback loop involving Fkh2 and Sep1 that may control expression dynamics in the G2/M phase of the fission yeast cell cycle.

Despite these successes, our analysis also showed certain limitations of learning biochemically motivated models from expression data alone. In particular, the learned regulators cannot be interpreted as transcription factors, but rather reflect more abstract regulatory functions, potentially carried out by multiple transcription factors. Several factors may contribute to this result, including the assumption of our regulation model that factor binding indicates direct activity, and the score used by the learning algorithm which favours the most parsimonious model that can explain the data, resulting in regulation schemes with a small number of "abstract" regulators. Thus, the model can fail to correctly separate between regulators when their activities are highly correlated (*e.g*. Ace2 and Swi5 in *S. cerevisiae*), or when their peak activities are concentrated in a narrow part of the cell cycle (*e.g*. PBF, MBF and Ace2 in *S. pombe*).

By integrating promoter sequences explicitly into the regulation model [19] (rather than in *post hoc *validation) we can overcome some of the limitations of the current approach. Such an integration can incorporate promoter composition as hard or soft constraints to the regulatory network structure, or it can iterate between learning of these two phases [20]. Such approaches may be able to achieve better integration of these different sources of data, leading to a more accurate and interpretable model of the regulatory network.

## Methods

### Expression data

We used expression data from [12]. We concatenated 3 time series (*Elutriation 1, Elutriation 2, Elutriation 3*) to generate one data set with 60 time points. Of the 405 genes reported as having cyclic expression in [12], we filtered out genes with more than two missing values, ending up with a set of 248 genes.

### Expression data pre-processing and derivation of transcription rates

We estimated transcription rates from the expression levels at consecutive time points as described in [15]. Briefly, since we lack measured mRNA degradation rates for *S. pombe*, we estimate the transcription rates using the naïve assumption that the minimal transcription rate for cycling genes during the cell cycle is zero. This biases our estimate of mRNA degradation rates to the low side. Note that using raw expression levels as inputs instead of rates would be equivalent to assuming infinite degradation rates. Running the ensemble learning on raw expression levels yields similar results with a noisier estimate of the affinity parameters. We therefore use the estimated transcription rates in the reported results.

### Regulation model

To model dependencies between a target gene's transcription rates and the (unknown) levels of its regulators, we used a model we previously developed based on the kinetics of binding and dissociation of transcription factors from their binding sites [15]. We considered up to two regulators (cooperative or non-cooperative) per target gene, allowing for either activation or repression. This limit on the number of regulators is driven from considerations of learnability and richness of representation: models with a higher number of regulators are richer in their representation power, but are also harder to learn uniquely from the available amount of data. The model is parameterized by the affinity parameters, *γ*_*i *_and the activity states of different regulator combinations, *α*_*I *_(Figure 2b). The transcription rate of gene *i *at time *t*, *tr*_*i*_*(t) *as a function of the activity of its regulators at that time, *r*_*i1*_*(t) *and *r*_*i2*_*(t)*, is modelled as:

(1)

where *ε*_*i*_*(t) *is a zero mean Gaussian noise variable, and *g *is the regulation function:

(2)

where *Z *is a normalizing partition function and *β*_*i *_is the maximal transcription rate of gene *i*. This family of models can describe different modes of regulation, using different combinations of *α*_*I *_parameters. These include activation, repression or a combination of one activator and one repressor; cooperative or redundant activation (akin to an AND or an OR gate, respectively), and even competitive activation (similar to a XOR gate). Model learning is feasible due to a "several to many" relation: a few regulators control the expression of many target genes using combinatorial regulation.

### Modularization

To reduce the number of parameters, the algorithm learned modular models (Figure 2d), where subsets of genes shared the same set of regulators and parameters. This greatly simplifies the model (at the cost of loss of some resolution). Once the modular model was learned, an additional iteration of parameter learning was applied without the modularization constraints, thus learning different kinetic parameters for each target gene.

### Structure learning

We used an iterative structure learning algorithm. The initial number of hidden regulators was set to K = 3,4,5 or 6. An initial connection topology between regulators and target genes was created using a linear sparse decomposition of the input data matrix using K components and 2 non-zero coefficients per target gene (see Additional file 2). The algorithm then iterated between two steps of optimization: (1) Regulation model and parameter learning and (2) gene assignment, similar to [5]:

**1. Regulation model and parameter learning **– For the current gene assignment, a search through model space was performed for the best module regulation model. This search consists of greedy hill-climbing steps, where in each step all topologies resulting from one of several possible actions are evaluated, and the highest scoring one is chosen. The possible actions are addition/removal of a connection between a regulator and a module; merging of two regulators; merging of two modules; and splitting of two modules. For each tested network topology, the regulation parameters of each module, {*γ*_*i*_, *α*_*I*_}, and the hidden regulator time profiles {}, were optimized using a constrained non-linear optimization algorithm.(*fmincon *in Matlab). The score used (BIC score) rewards for data fitting while penalizing for model complexity.

**2. Gene assignment **– For the current regulator profiles and module regulation parameters, each gene *i *was assigned to the module whose parameters fitted the data *tr*_*i*_*(t), t = 1..T *(using Eq. 1 for) with the lowest error.

The algorithm terminates when there are no more changes to gene assignment.

### Bootstrapping

To estimate our confidence in different features of the model, we learned an ensemble of 90 models (Figure 2e). For each model, a subset of 200 genes was randomly sampled from the 248 target genes set. The transcription rate time series of these 200 genes were input to the structure learning algorithm described above, resulting in one parameterized model.

### Identification of core modules

To analyze the results of the ensemble of runs, we defined a set of core target gene modules in the following way: we computed the module co-occurrence matrix *C*, in which *C*_*ij *_is the fraction of runs in which genes *i *and *j *were placed in the same module. We then hierarchically clustered the rows of *C *using average linkage agglomerative clustering (UPGMA) with a Euclidian distance metric. Each internal node in the clustering tree defines a subset of genes. For each such subset, we computed the mean (*μ*_*frac*_) and standard deviation (*σ*_*frac*_) over all runs of the fraction of the subset co-occurring in a module. We looked for the set of internal nodes comprising the highest cut in the tree for which *μ*_*frac *_- *σ*_*frac *_> 0.5. This set defined the core target gene modules. Note that this definition yielded consistent results when the number of genes in each run *G *or the number of regulators *K *was changed. We subsequently assigned several additional genes of interest (not included in the 248 input genes due to missing values) to modules, based on the similarity of their expression patterns to that of module genes. These genes, manually chosen based on their known functionality in the cell cycle, include cell cycle related regulators (Res2, Cdc10, Ace2, Res1 and Rep2) and kinases (Cig2, Plo1, and Rep2).

### Regulator mapping

The regulators learned in each run are anonymous (*i.e*. have no known identity). To interpret the ensemble of runs, we mapped the regulators between the different runs using two distinct methods. The methods yielded consistent results. In the first approach, we named the regulators in each run using an iterative clustering method based on the similarity of the learned regulator profiles (Figure 2e, right panel). The regulator names were initialized randomly. We then cycled through the runs and in each run we assigned each regulator to the group with the highest mean similarity to its profile. This was repeated until no change in assignment occurred. In the second approach, we clustered the columns of the affinity matrix *A *between the regulators to core modules. Each row in *A *represents a core module *m*, and each column represents one regulator in a particular run.

### Selection of number of regulators

We have run the ensemble learning method with different numbers of regulators (*K *= 3, 4, 5, 6). In the ensembles initialized with more than four regulators, one or more of the regulators usually ended up degenerate (*i.e*. not connected to any module in a significant number of runs). Moreover, the resulting division to core modules was highly similar to that obtained with *K *= 4. We therefore present results from ensembles with four regulators.

### Analysis of module gene content

To analyze the functions of the target genes in each module, we used the Gene Ontology (GO) annotations for fission yeast genes [21]. Since many of these genes were not annotated, we also examined the GO annotations of their budding yeast orthologs (orthologs were determined as in [22]).

### Generation of motif library

We learned *cis*-regulatory motifs from the 1000 bp promoter regions of the target gene sets. (Promoters in intergenic regions of less than 1000 bp were cropped accordingly.) We used two definitions for gene sets for this procedure: members of a core module, or all targets of a regulator (across modules). The former results in a group of correlated genes; the latter is a principled approach to learn the binding site of a regulator. We used four algorithms to search for motifs (AlignACE [23], MDscan [24] and Meme [25], as implemented in [26]; and SeedSearcher [27]) The resulting motifs were clustered as in [28] and representatives of each cluster were chosen to reduce redundancy. Known motifs were identified and named by their similarity to previously characterized motifs from *S. pombe *or *S. cerevisiae*. We supplemented this motif library with five other *S. cerevisiae *motifs related to the cell cycle, which do not have a known counterpart in *S. pombe *(YHP1, YOX1, ASH1, FHL1 and MCM1), resulting in a library of 26 motifs. None of the *S. cerevisiae *motifs was enriched in subsequent analysis (below).

### Scanning promoters for motifs

We scanned the 1000 bp promoters of all the fission yeast genes for appearances of the 26 motifs using a P < 0.05 score cutoff. We then computed enrichments (using the hypergeometric distribution, with cutoff at P < 0.05 or P < 0.005) for each of the motifs in each of the gene sets defined by either module members or targets of a putative regulator.

## Authors' contributions

IN and AR perceived and designed the research. IN designed and wrote the algorithms, and performed the analysis. AR contributed to algorithm design and biological analysis. Both authors wrote the article and approved the final manuscript.

## Supplementary Material

Additional file 1 **Table S1. Gene composition of the 16 core modules**. The full gene list of each of the 16 learned core transcriptional modules. For each of the member genes, whenever a clear orthologous gene was found in *S. cerevisiae *[22], the annotation of that gene is shown too. Genes shown in italics are cell cycle regulators that did not participate in the model learning (due to missing values), and were later associated to one of the modules based on expression profile similarity. Click here for file

Additional file 2 **Supplementary Note. Analysis of the regulatory model learning method**. Description of supplemental experiments and analysis. Click here for file

Additional file 3 **Table S2. Binding motifs enriched in modules**. A non-redundant library of *cis*-regulatory motifs found using our pipeline, for which at least one of the gene modules is significantly enriched. Shown are the sequence logo for each motif, the name of a matching known motif (if available), the source of the motif (M – module, R – regulator; see text) and all significant associations between a motif and a module. The format for each such entry is: Top – number of genes in module with the motif/number of genes in module without the motif/number of genes in the genome outside the module with the motif. Bottom – enrichment *p*-value. Enrichments were computed for two PSSM score cutoffs (P < 0.05 or P < 0.005), and the best result is reported. Click here for file

Additional file 4 **Figure S1. Transcription rate profiles of the 16 core modules**. Shown are the transcription rate time profiles for members of each of the 16 core modules in the unified model. The boundaries of M, G1, and S phases for the middle part of the series (corresponding to Elutriation 2 experiment) are shown with red dashed lines, as in Figure 3. Click here for file

Additional file 5 **Figure S2. Expression profiles for K-means clustering, K = 12**. Shown are the expression profiles of genes in each of the 12 clusters obtained in the K-means run. Previously known cell cycle motifs (FKH, PCB, MCB, Histone box) that were found to be significantly enriched in a particular cluster are shown, along with the number of genes in the cluster with the motif/number of genes in the cluster without the motif. Click here for file

Additional file 6 **Figure S3. Expression profiles for K-means clustering, K = 16**. As in Figure S2. Click here for file

Additional file 7 **Figure S4. Expression profiles for K-means clustering, K = 5**. As in Figure S2. Click here for file

Additional file 8 **Figure S5. Core modules and regulators resulting from the initial sparse decomposition in the 90 runs**. Shown are transcription rate profiles of the 5 core modules and inferred activity profiles of the four regulators in the unified model resulting from the initial sparse decomposition in each of the 90 ensemble runs. For the regulator profiles, mean and one standard deviation curves are shown. Click here for file

Additional file 9 **Figure S6. Network modules and learned regulator profiles resulting from a single run with all 248 genes**. As in Figure S5. Click here for file

Additional file 10 **Figure S7. Network modules and learned regulator profiles resulting from the initial sparse decomposition of a single run with all 248 genes**. As in Figure S5. Click here for file
