# Supplementary material for: BRNI: Modular analysis of transcriptional regulatory programs
Source: BMC Bioinformatics. 2009 May 20;10:155. doi: 10.1186/1471-2105-10-155 (PMC2694189; doi:10.1186/1471-2105-10-155)
Supplement: Additional file 2 — Supplementary Note. Analysis of the regulatory model learning method. Description of supplemental experiments and analysis. [file 1471-2105-10-155-S2.pdf]

Supplementary Note: Analysis of the regulatory model learning method

In this paper we present a biologically-motivated method for learning an expression based regulation model. Our approach is based on an earlier method [14] ,with two important modifications: **(1)** gene modularization (associated with a new model learning algorithm); and **(2)** ensemble learning. Here we try to analyze the contribution of each of these two elements separately, by examining results from the following model learning/gene clustering methods on the data set used in the paper:

1. Initial linear sparse decomposition of the full 248 gene set.
2. The final model from a network learning run with the full 248 gene set
3. The initial sparse decompositions from each of the 90 runs in our ensemble, each using a subset of 200 genes.
4. The full ensemble run reported in the paper
5. K-means clustering with K=5, 12 or 16, using expression profile correlation as a similarity measure.

The chart below shows how methods 1-4 relate to each other:

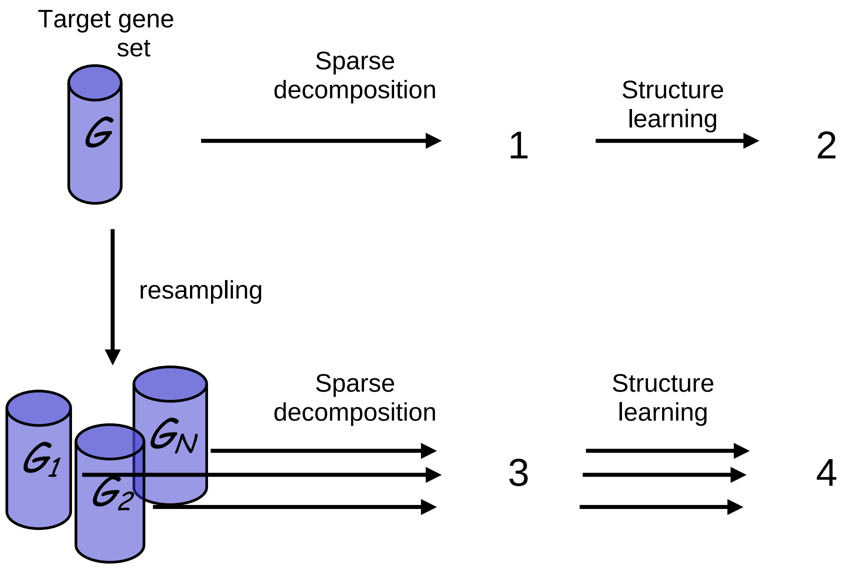

Comparing **method 1** with **2** and **method 3** with **4** can be informative about the contribution of the structure learning step, with or without ensemble learning, respectively. Comparing **method 1** with **3** and **method 2** with **4** can be informative about the contribution of the ensemble learning in the context of a simple

decomposition or full structure learning, respectively. The **K-means** runs serve as an external “yardstick” for comparison, where the *K* values were chosen to be similar to the module numbers obtained in tests 1-4 above.

In our runs, the initial sparse decomposition to *K* components, with at most 2 “regulatory” connections is done in the following way: we start by singular value decomposition (SVD) of the data, retaining *K* components, and then for every target gene, zeroing out all but the strongest 2 connections. Modules (or clusters) are defined in this context as groups of genes sharing the same set of regulators (or components).

We compare these different runs in two aspects: **(1)** Coherence of the resulting clusters: How tight and correlated are the genes in each cluster? Are the clusters over-fragmented or too coarse? and **(2)** Correspondence of the clusters with known *S. pombe* motifs: Are these motifs significantly enriched in some of the clusters? In cases they are, do they “hit” a large fraction of the genes in that cluster? Note that we cannot use the full motif library we learned for this test, since it was learned from gene sets defined by method 4 above, and would therefore present results biased in favor of this run. Moreover, good correspondence of a method with known motifs provides motivation for using that method as a basis for discovering novel motifs. We therefore relied on known motifs for this assessment (Fkh, MCB, PCB, Histone box, Ace2). **Figures S1** through **S7** show the clusters for each of those runs, denoting for each cluster motifs which were significantly over-represented in it, in cases there were any. Below is a summary of this comparison:

Method	Number of clusters	Mean correlation within clusters	Enrichments for known motifs in any module (positive/negative)				
			ACE2	PCB	FKH	MCB	Histone
**(4)** ensemble, final modules	16	0.66	6/6, 3/14;	7/4	5/6	3/6	4/0
**(3)** ensemble initial decompositions	5	0.12	9/22	24/142	52/114	9/157	10/156
**(2)** single run, final modules	12	0.63	9/17	8/10	7/11		3/3
**(1)** single run, initial decomposition	5	0.15	9/21	21/134	47/108	9/146	9/146
K-means, *K*=12	12	0.61	10/6, 9/11				
K-means, *K*=16	16	0.68	9/22	9/16	15/10		5/15
K-means, *K*=5	5	0.54	10/60	11/32	18/25	5/65	5/26

Enrichment info is shown for any module with significant enrichment, in the format: number of genes in module with the motif/number of genes in module without the motif.

Method 1 and **3** are based on linear sparse decomposition of the input data (Figure S5, S7). These seem to under-cluster the data: the typical sparse decomposition yields only 5 clusters, where one of them is dominant (containing >60% of the genes). Even the smaller clusters seem less tight than the clusters obtained in **methods 2** and **4 (Figure S1, S6)**. Furthermore, known motifs only hit small fractions in each cluster in these initial decompositions, suggesting that these runs provide too coarse a resolution, or simply miss-clustering of the genes. Notably, the large cluster hits 4 of the 5 motifs. The **K-means** run with *K*=5, though more balanced in cluster size, still presents under-clustering as reflected by motif enrichment (Figure S4). The **structure learning** step that follows the sparse decomposition in **methods 2** and **4** greatly refines the module (cluster) structure compared to the initial sparse decomposition: several steps of module splitting and rearrangement break down the large module, and the resulting module organization is more balanced and coherent.

The core module definition of the **ensemble** run, requiring two genes to be grouped together in a majority of runs in order to be considered in the same module, leads to further refinement of some of the modules, generating modules which are more uniform with respect to known motifs. This is evident from comparing **method 2** with **4** (**Figure S1** and **S6**, **Table 2**). For example, the ensemble leads to a “clean” Histone module (all 7 genes have the Histone motif), while in the single run we get a mixed module (only 3 out of 6 genes have the Histone motif). Similarly, the FKH, PCB and ACE2 motifs are all present at a higher fraction of module genes in the ensemble run than in the single run.

Comparing **method 2** or **4** with the **K-means** runs with the same number of clusters (Figure S3, S4) shows that **K-means** clusters which are significantly enriched for known motifs generally show a smaller fraction of hit genes, with some exceptions (*e.g.* ACE2 in the K=12 run). Moreover, in the K=12 run only two of the five motifs are significantly enriched in any of the clusters.

In summary, both the ensemble learning and the structure learning components of the BRNI method contribute to purifying/sharpening the modules, as reflected in module coherence and in enriched motif signals.
