# Supplementary figures and images for: BRNI: Modular analysis of transcriptional regulatory programs
Source: BMC Bioinformatics. 2009 May 20;10:155. doi: 10.1186/1471-2105-10-155 (PMC2694189; doi:10.1186/1471-2105-10-155)

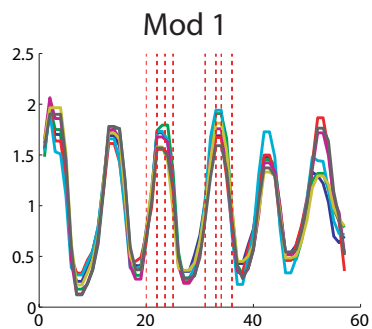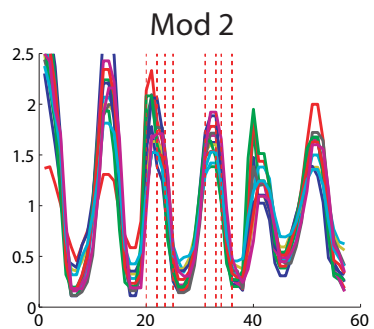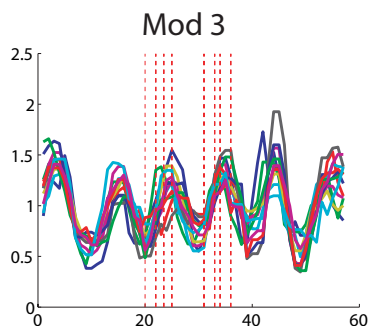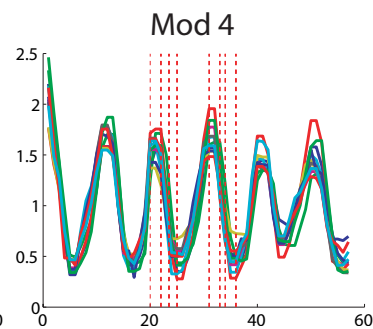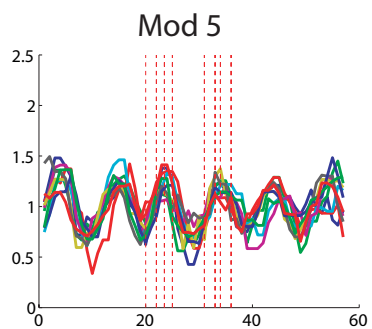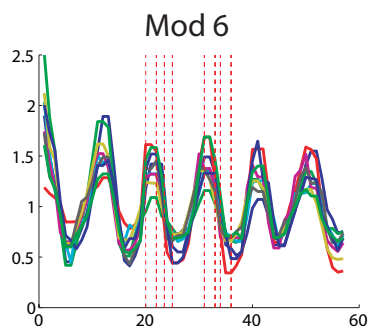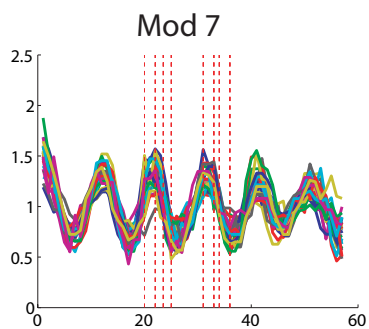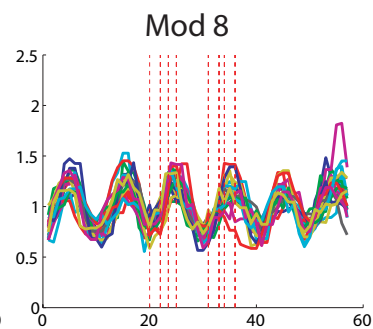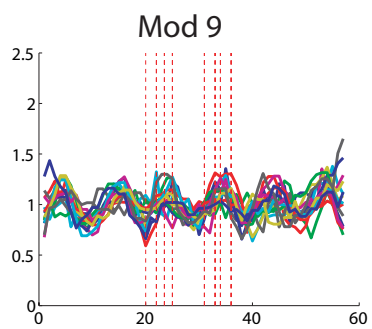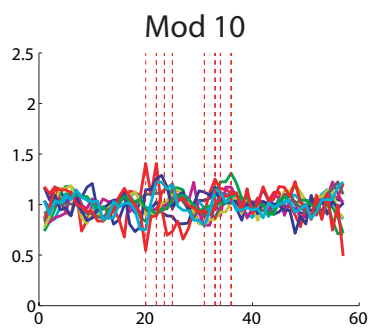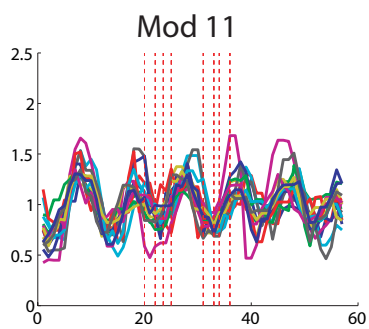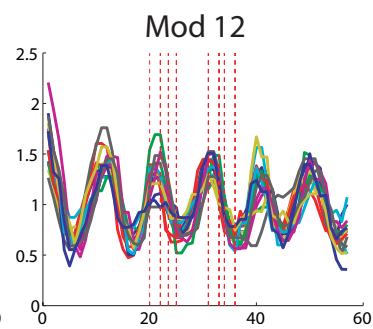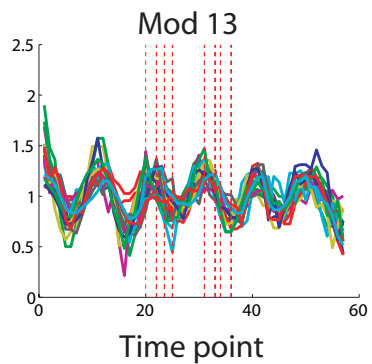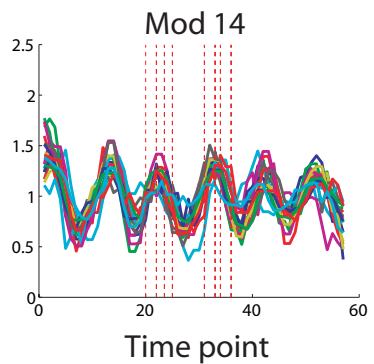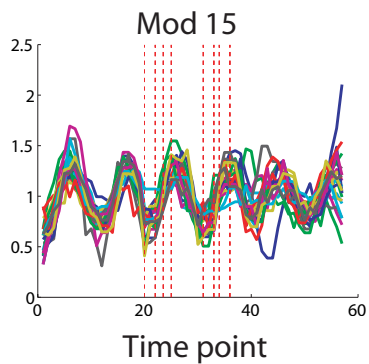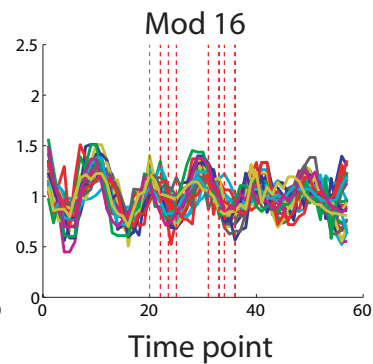

Supplement: Additional file 4 — Figure S1. Transcription rate profiles of the 16 core modules. Shown are the transcription rate time profiles for members of each of the 16 core modules in the unified model. The boundaries of M, G1, and S phases for the middle part of the series (corresponding to Elutriation 2 experiment) are shown with red dashed lines, as in Figure 3. [file 1471-2105-10-155-S4.pdf]

K-means clustering, K=12

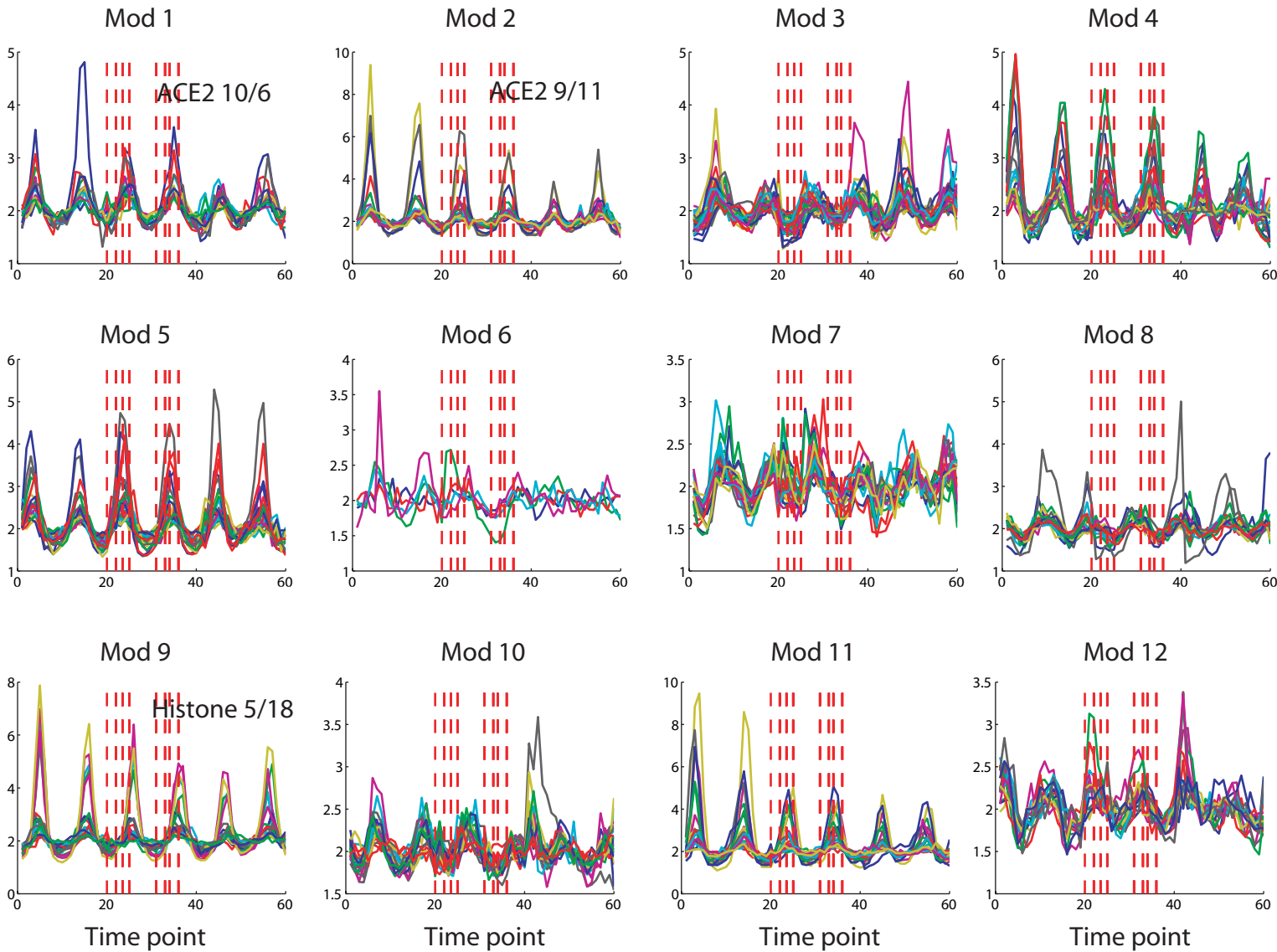

Supplement: Additional file 5 — Figure S2. Expression profiles for K-means clustering, K = 12. Shown are the expression profiles of genes in each of the 12 clusters obtained in the K-means run. Previously known cell cycle motifs (FKH, PCB, MCB, Histone box) that were found to be significantly enriched in a particular cluster are shown, along with the number of genes in the cluster with the motif/number of genes in the cluster without the motif. [file 1471-2105-10-155-S5.pdf]

# K-means clustering, K=16

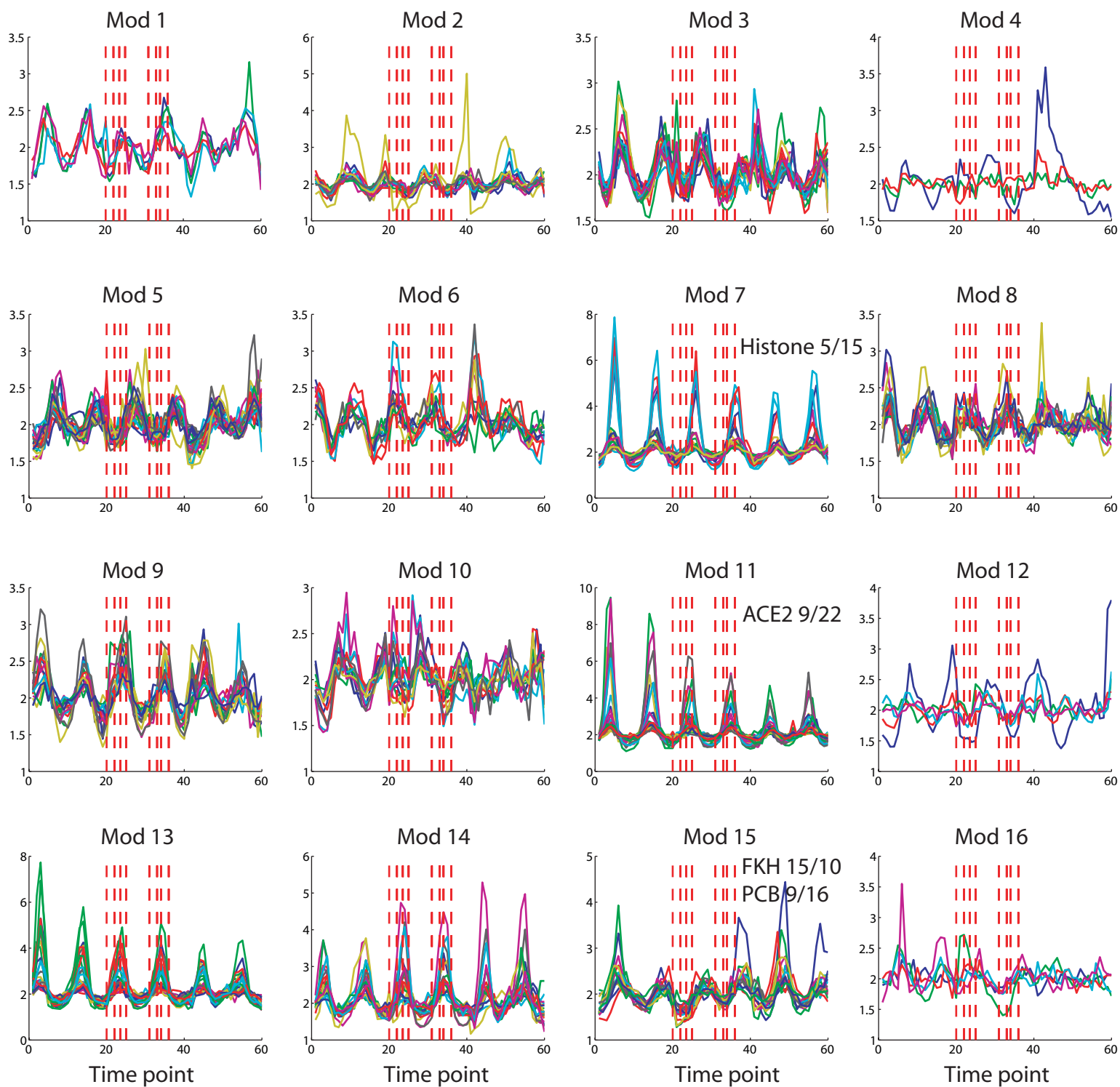

Supplement: Additional file 6 — Figure S3. Expression profiles for K-means clustering, K = 16. As in Figure S2. [file 1471-2105-10-155-S6.pdf]

K-means clustering, K=5

Mod 1

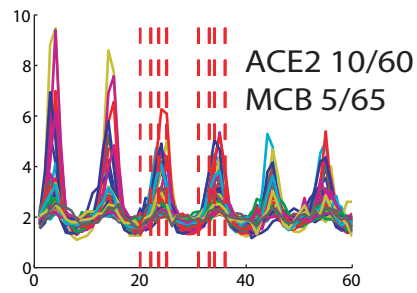

Mod 2

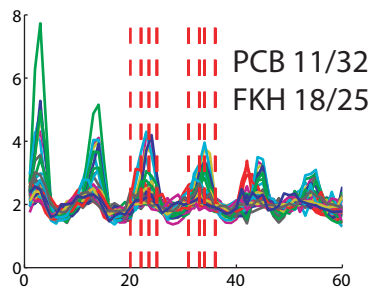

Mod 3

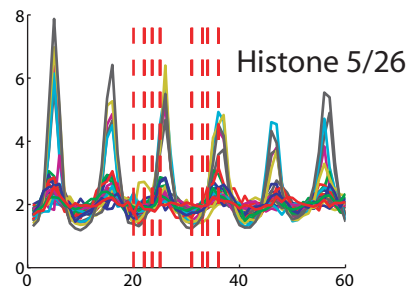

Mod 4

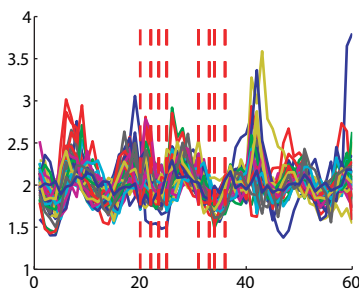

Mod 5

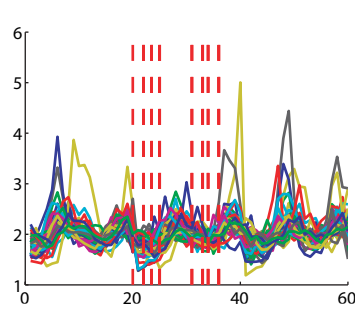

Supplement: Additional file 7 — Figure S4. Expression profiles for K-means clustering, K = 5. As in Figure S2. [file 1471-2105-10-155-S7.pdf]

Ensemble of 90 runs, initial sparse decomposition

Modules

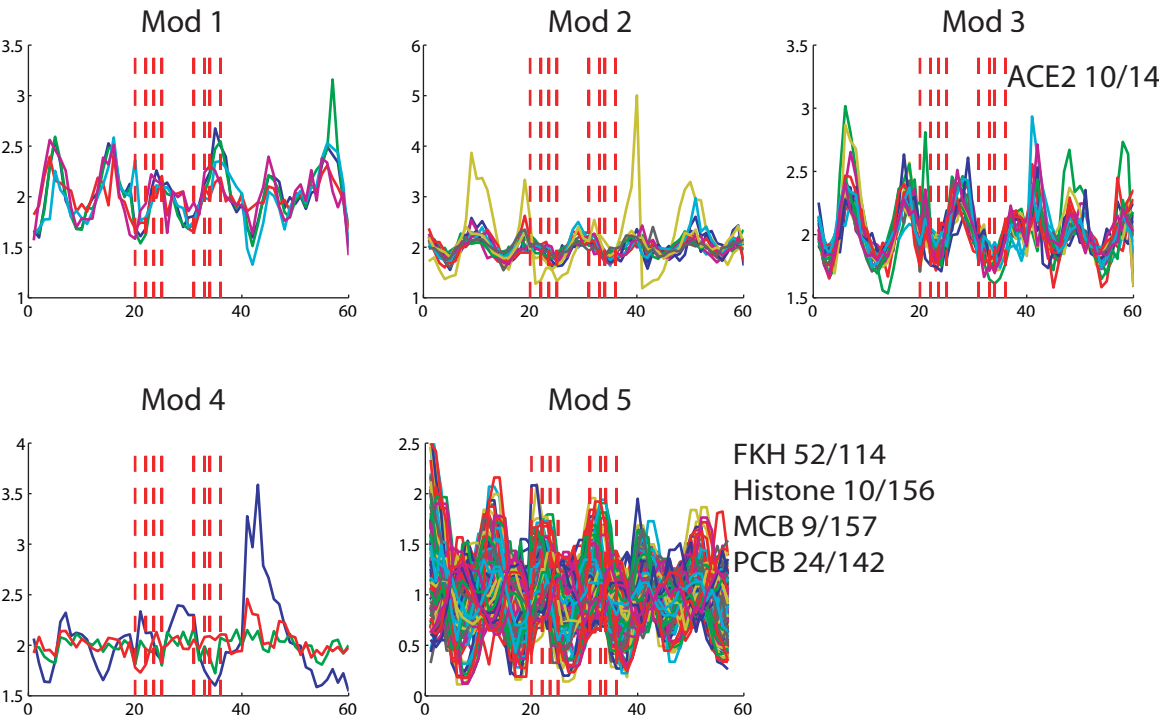

Regulators

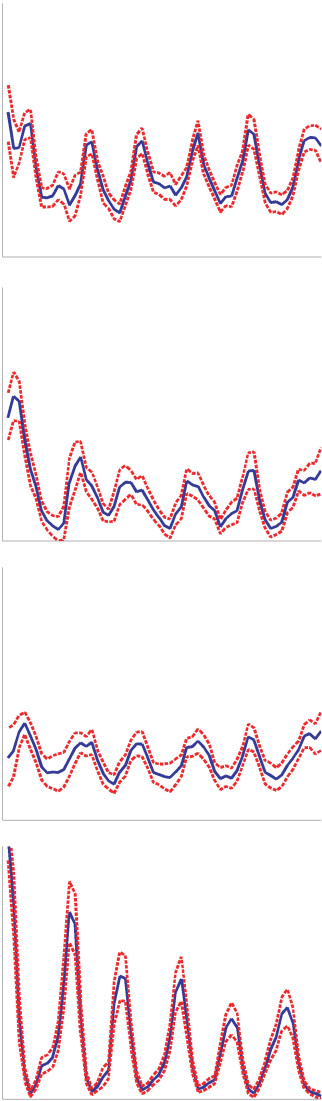

Supplement: Additional file 8 — Figure S5. Core modules and regulators resulting from the initial sparse decomposition in the 90 runs. Shown are transcription rate profiles of the 5 core modules and inferred activity profiles of the four regulators in the unified model resulting from the initial sparse decomposition in each of the 90 ensemble runs. For the regulator profiles, mean and one standard deviation curves are shown. [file 1471-2105-10-155-S8.pdf]

# Single run with all 248 genes

## Modules

Mod 1

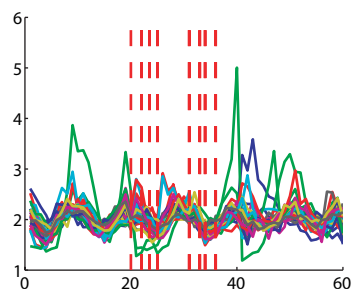

Mod 2

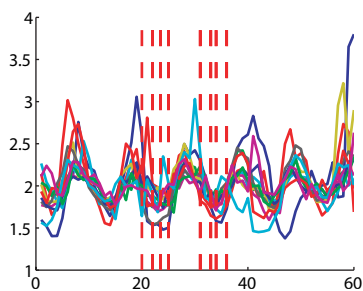

Mod 3

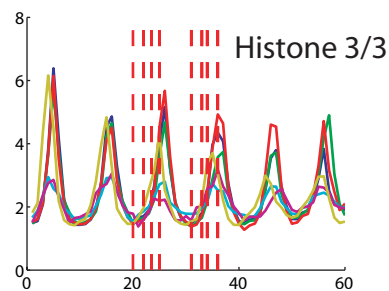

Mod 4

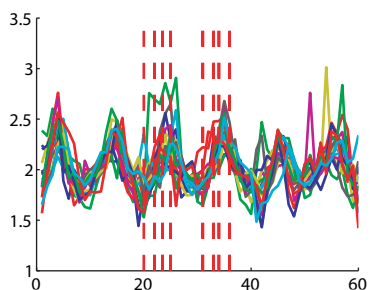

Mod 5

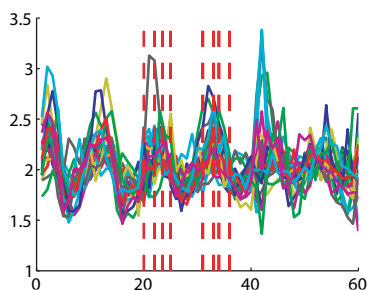

Mod 6

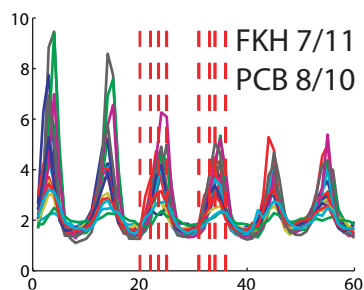

Mod 7

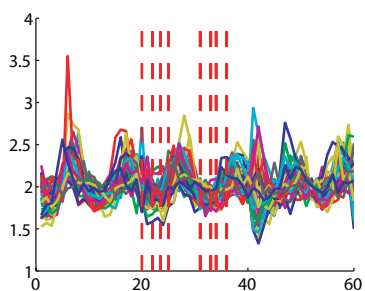

Mod 8

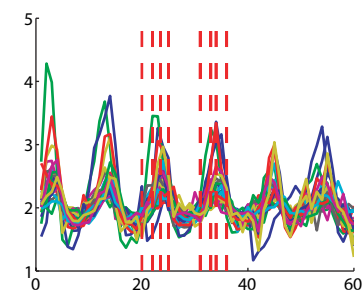

Mod 9

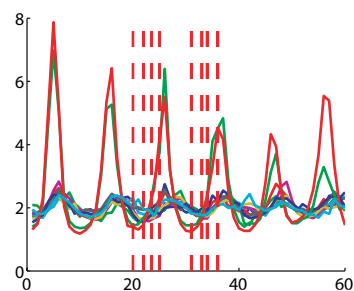

Mod 10

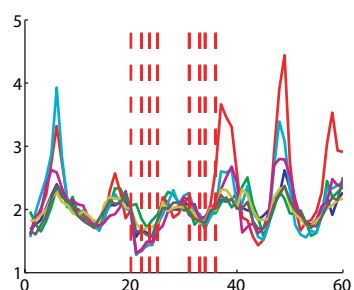

Mod 11

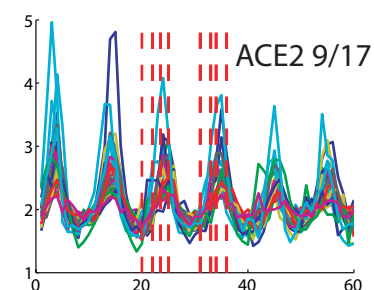

Mod 12

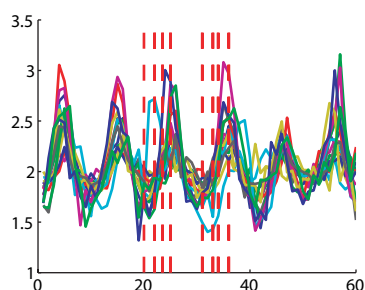

## Regulators

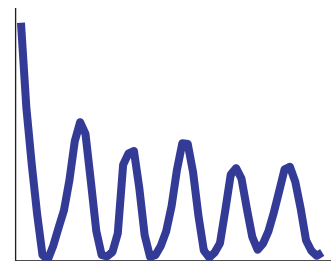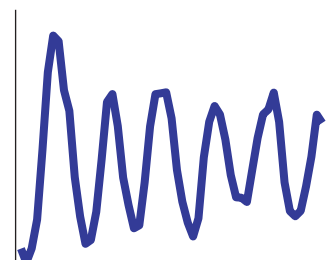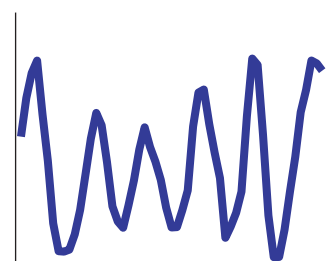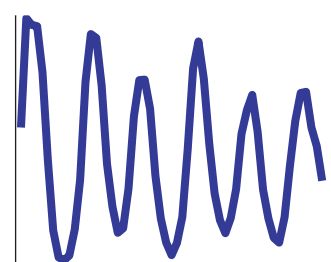

Supplement: Additional file 9 — Figure S6. Network modules and learned regulator profiles resulting from a single run with all 248 genes. As in Figure S5. [file 1471-2105-10-155-S9.pdf]

Single run with all 248 genes, initial sparse decomposition

Modules

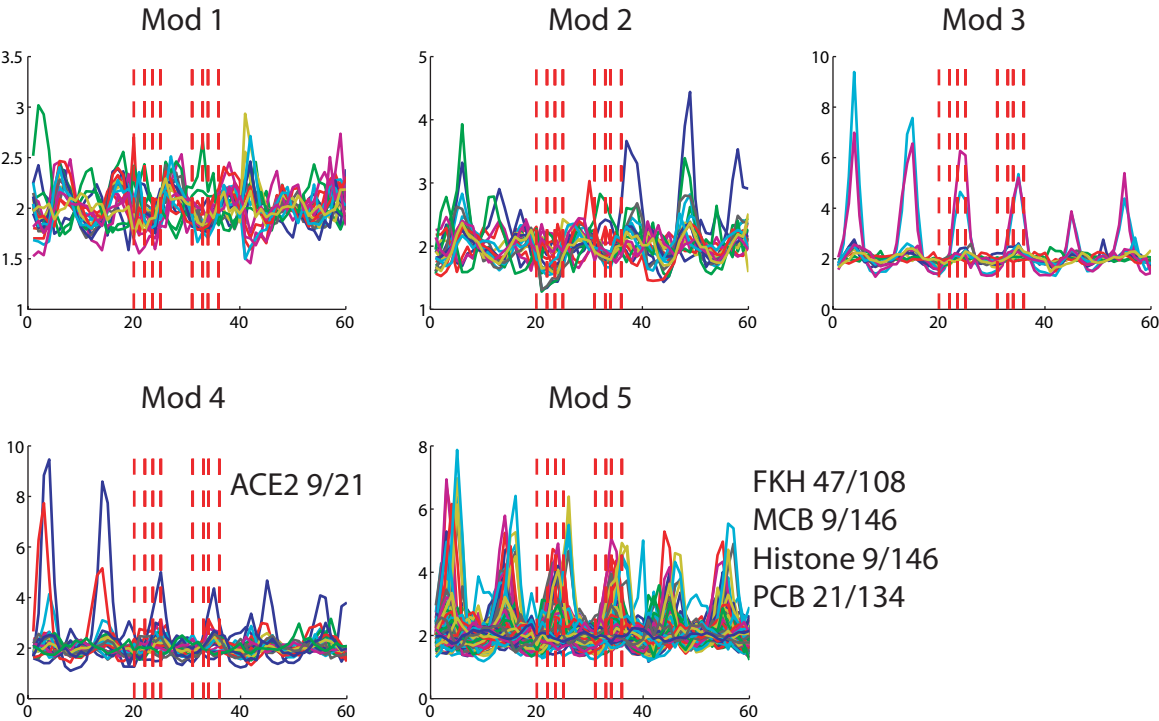

Regulators

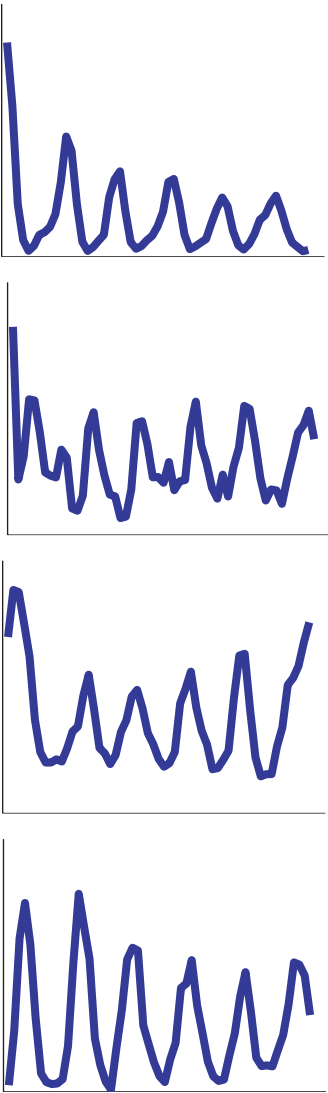

Supplement: Additional file 10 — Figure S7. Network modules and learned regulator profiles resulting from the initial sparse decomposition of a single run with all 248 genes. As in Figure S5. [file 1471-2105-10-155-S10.pdf]
