# Supplementary material for: BRNI: Modular analysis of transcriptional regulatory programs
Source: BMC Bioinformatics. 2009 May 20;10:155. doi: 10.1186/1471-2105-10-155 (PMC2694189; doi:10.1186/1471-2105-10-155)
Supplement: Additional file 3 — Table S2. Binding motifs enriched in modules. A non-redundant library of cis-regulatory motifs found using our pipeline, for which at least one of the gene modules is significantly enriched. Shown are the sequence logo for each motif, the name of a matching known motif (if available), the source of the motif (M – module, R – regulator; see text) and all significant associations between a motif and a module. The format for each such entry is: Top – number of genes in module with the motif/number of genes in module without the motif/number of genes in the genome outside the module with the motif. Bottom – enrichment p-value. Enrichments were computed for two PSSM score cutoffs (P < 0.05 or P < 0.005), and the best result is reported. [file 1471-2105-10-155-S3.pdf]

Name	Source	Logo	mod1	mod2	mod4	mod6	mod7	mod8	mod14
Ace2	M	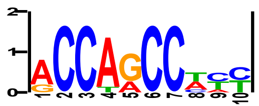		6/6/44<br>6.21e-10			13/14/354<br>1.04e-08		
	M	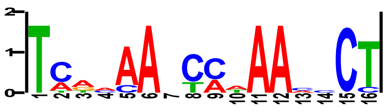	4/0/288<br>1.12e-05						8/10/287<br>3.32e-06
	M	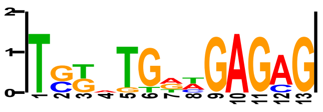							4/14/31<br>5.59e-06
	M	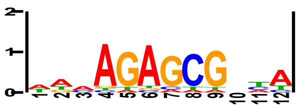						11/9/256<br>8.34e-10	
	R	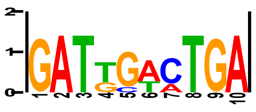	3/1/35<br>1.57e-06						
	M	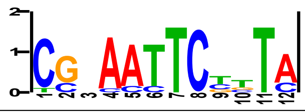							8/10/343<br>1.21e-05
	M	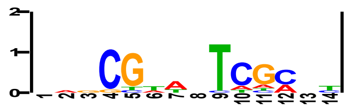							9/9/226<br>3.06e-08
	M	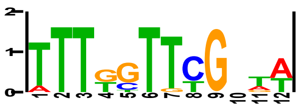						8/12/275<br>6.28e-06	
PCB	M	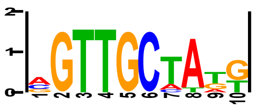			7/4/234<br>1.48e-07				
Fkh2 (FLEX)	M	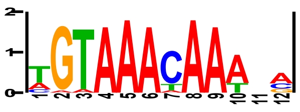			5/6/209<br>4.96e-05				
MCB	R	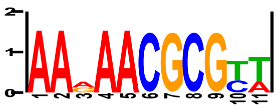				3/6/31<br>2.3e-05			
	R	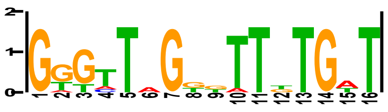	4/0/333<br>1.99e-05						
	R	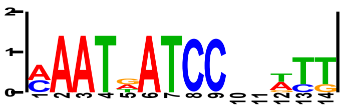	3/1/76<br>1.47e-05	9/3/288<br>1.45e-09					
	M	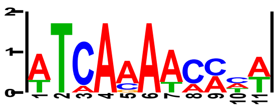	4/0/216<br>3.60e-6						
	M	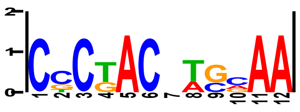	3/1/17<br>2.13e-07						
	M	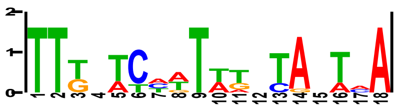	4/0/25<br>8.84e-10						
ACCCT box	M	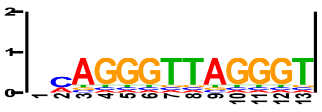	4/0/61<br>2.52e-08						
	M	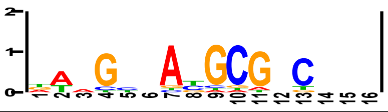		6/6/29<br>6.46e-11					
